# Multiphase Dual-Energy Spectral CT-Based Deep Learning Method for the Noninvasive Prediction of Head and Neck Lymph Nodes Metastasis in Patients With Papillary Thyroid Cancer

**DOI:** 10.3389/fonc.2022.869895

**Published:** 2022-04-20

**Authors:** Dan Jin, Xiaoqiong Ni, Xiaodong Zhang, Hongkun Yin, Huiling Zhang, Liang Xu, Rui Wang, Guohua Fan

**Affiliations:** ^1^ Department of Radiology, Second Affiliated Hospital of Soochow University, Suzhou, China; ^2^ Department of Advanced Research, Infervision Medical Technology Co., Ltd, Beijing, China

**Keywords:** thyroid cancer, dual-energy CT (DECT), lymph nodes metastasis, multiphase, deep learning

## Abstract

**Purpose:**

To develop deep learning (DL) models based on multiphase dual-energy spectral CT for predicting lymph nodes metastasis preoperatively and noninvasively in papillary thyroid cancer patients.

**Methods:**

A total of 293 lymph nodes from 78 papillary thyroid cancer patients who underwent dual-energy spectral CT before lymphadenectomy were enrolled in this retrospective study. The lymph nodes were randomly divided into a development set and an independent testing set following a 4:1 ratio. Four single-modality DL models based on CT-A model, CT-V model, Iodine-A model and Iodine-V model and a multichannel DL model incorporating all modalities (Combined model) were proposed for the prediction of lymph nodes metastasis. A CT-feature model was also built on the selected CT image features. The model performance was evaluated with respect to discrimination, calibration and clinical usefulness. In addition, the diagnostic performance of the Combined model was also compared with four radiologists in the independent test set.

**Results:**

The AUCs of the CT-A, CT-V, Iodine-A, Iodine-V and CT-feature models were 0.865, 0.849, 0.791, 0.785 and 0.746 in the development set and 0.830, 0.822, 0.744, 0.739 and 0.732 in the testing set. The Combined model had outperformed the other models and achieved the best performance with AUCs yielding 0.890 in the development set and 0.865 in the independent testing set. The Combined model showed good calibration, and the decision curve analysis demonstrated that the net benefit of the Combined model was higher than that of the other models across the majority of threshold probabilities. The Combined model also showed noninferior diagnostic capability compared with the senior radiologists and significantly outperformed the junior radiologists, and the interobserver agreement of junior radiologists was also improved after artificial intelligence assistance.

**Conclusion:**

The Combined model integrating both CT images and iodine maps of the arterial and venous phases showed good performance in predicting lymph nodes metastasis in papillary thyroid cancer patients, which could facilitate clinical decision-making.

## Introduction

Approximately 30%-80% of patients with papillary thyroid cancer (PTC) have cervical lymph nodes (LNs) metastasis, especially to the lateral neck (1, 2). Recognizing the presence of metastatic LNs is pivotal for determining a correct therapeutic strategy for patients and is beneficial to the clinical command and prognosis evaluation of PTC. At present, there are many ways to evaluate the benign and malignant status of LNs in patients with PTC. Although the 2015 American Thyroid Association (ATA) guidelines consider preoperative ultrasonography (US) the preferred technique for the assessment of LNs status in PTC patients (3), this technique has deficiencies when evaluating LNs at low cervical levels, such as the retropharyngeal area and upper mediastina, and depends heavily on the clinical experience of the operator. Computed tomography (CT) can compensate for the above partial defects by relying on excellent tissue and spatial resolution. However, the subjective nature of morphologic criteria such as node size, degree and pattern of enhancement, necrosis, and extranodal extension for visually evaluating whether cervical LNs are benign or metastatic results in diminished reproducibility and objectivity, especially for small LNs without specific morphological features.

With the widespread application of dual-energy spectral CT (DECT) called gemstone spectral imaging (GSI), some studies have found that CT image features with higher spatial resolution and energy spectrum parameters provided by DECT can be conducive to the detection and evaluation of LNs status in PTC (4). Several studies have suggested that the slope of the spectral Hounsfield unit curve (λ^HU^), the normalized iodine concentration (NIC), and the normalized effective atomic number are effective parameters for diagnosing LNs metastasis in patients with PTC (1, 4, 5)

In recent years, artificial intelligence (AI) based on deep learning (DL) has been a frontier computational method that simulates brain structures connecting a large number of neurons, can complete image-recognition tasks in a short time without subjective assessment and includes nonvisual image details, which have a high-dimensional association with clinical issues (6–8). Theoretically, the combination of dual-energy CT and deep learning methods may potentially improve the preoperative predictive performance for LNs metastasis in patients with PTC.

The purpose of our study was to investigate the usefulness of DL models based on multiphase DECT for predicting LNs metastasis in patients with PTC and to compare them with radiologists’ assessments.

## Materials and Methods

### Patient Enrollment

The study was approved by the institutional review board of our hospital, and the requirement for informed consent was waived. Dual-energy spectral CT images of papillary thyroid cancer patients from April 2018 to December 2020 were retrospectively collected. The inclusion criteria were as follows: 1) preoperative dual-energy CT was performed within two weeks before surgery; 2) patients underwent lymphadenectomy, and the tumor metastasis of lymph nodes was pathologically confirmed. The exclusion criteria were as follows: 1) patients had received any anti-cancer treatment before surgery; 2) patients had suffered from other cancer at the same time; 3) low CT image quality or lymph nodes less than 5mm.

**Figure 1:**
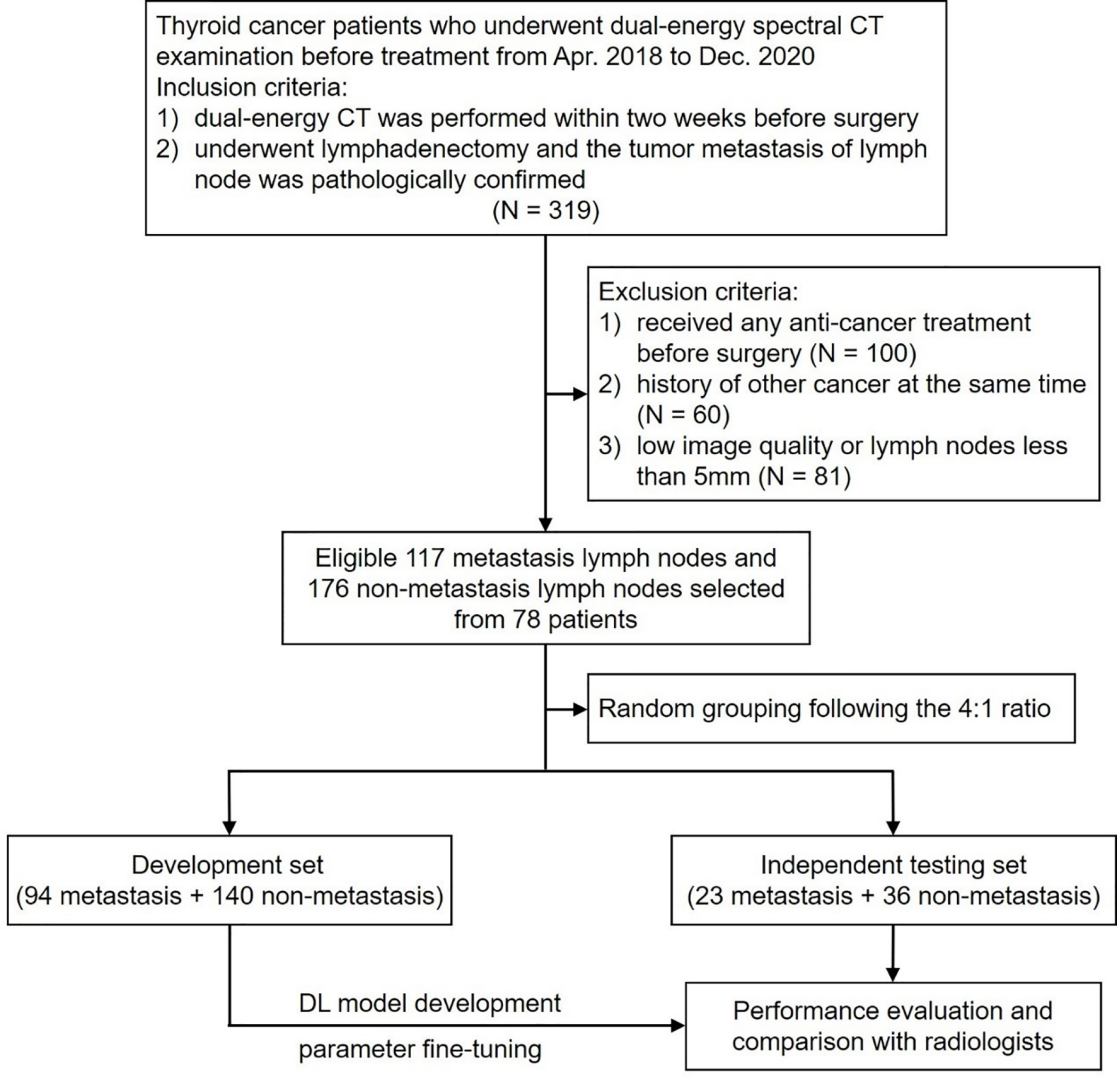
Flowchart of patient enrollment and study design.

Ultimately, a total of 117 lymph nodes with tumor metastasis and 176 lymph nodes without tumor metastasis from 78 patients were enrolled in this study. The patient enrollment flowchart is shown in **Figure 1**. These lymph nodes were divided into a development set (140 nonmetastatic and 94 metastatic) and an independent testing set (36 nonmetastatic and 23 metastatic) at a ratio of 4:1 using computer-generated random numbers.

### Acquisition of CT Images

All patients underwent dual-energy CT examinations in gemstone spectral imaging (GSI) mode (GE Discovery CT750 HD scanner; GE Healthcare, Princeton, NJ, USA). DECT scans were from the skull base to the aortic arch level and in the head-foot direction. The DECT scan parameters were as follows: 64×0.625-mm detector collimation; 0.8-sec tube rotation time; 0.984 pitch; 1.25-mm-thick sections; 1.5-mm-thick section increments; rapid switching of high (140 kVp) and low (80 kVp) tube voltages; 360-mA tube current. For contrast-enhanced scans, patients were injected with 1.6 mL/kg of nonionic iodinated contrast medium (300 mg I/mL) by a pump injector at a rate of 3.5 mL/s into the antecubital vein. Images of arterial and venous phases were performed after 25- and 55-sec delays, respectively.

Iodine maps of both arterial and venous phases at a 1.5-mm slice thickness can be autogenerated by DECT.

### Pathological Diagnosis of Lymph Nodes Metastasis

In this study, we adopted the labeling method of LNs imaging and pathological subregion comparison proposed by Park et al. (9). According to the LNs location standard established by the American Joint Committee on Cancer (AJCC), cervical LNs are divided into I-VII regions (10). The final histopathologic reports of the surgical neck dissection samples served as the reference standard for nodal metastasis. After obtaining the numbers of metastatic and nonmetastatic LNs in each region, DECT images were compared by a radiologist with 10 years of experience in head and neck radiology. If pathological results showed metastasis in all LNs in a subregion, the LNs seen in the image area were marked as metastatic LNs. If the pathological results showed no metastasis in any of the LNs in a subregion, the LNs seen in the imaging region were marked as nonmetastatic LNs. In addition, if there were both metastatic and nonmetastatic LNs in the subregion, the levels with mixed LNs were ruled out in further research.

### Qualitative Analysis of Lymph Nodes and Development of the CT-Feature Model

Eight morphological CT features of the lymph nodes from CT images were analyzed by two radiologists with 12 and 10 years of experience in head and neck imaging, including size, shape, margin, degree of enhancement, pattern of enhancement, calcification, cystic change, and extra-nodal extension. Size was determined by using the maximal short axis diameter. Degree of enhancement was assessed based on the neighboring muscle. Uniformity of enhancement was evaluated based on the arterial phase. Fuzzy boundaries and/or invasion into contiguous structures were deemed as extra-nodal extension (11). A third senior radiologist with more than 10 years of experience was consulted for the final decision if disagreements occurred. All radiologists were not aware of the pathological results.

The differences of CT image features were assessed in the development set through multivariate analysis using stepwise selection logistic regression. Only the CT image features with p < 0.05 were selected to build the CT-feature model.

### Segmentation of Lymph Nodes

The lymph nodes were manually segmented on both arterial phase and venous phase CT images, and the iodine maps shared the same segmentations with the corresponding CT images. Three-dimensional segmentation was performed by a radiologist with more than 10 years of experience, and the region of interest (ROI) was manually drawn freehand strictly within the border of lymph nodes on each slice of the CT images using ITK-SNAP software (v3.8.0, http://www.itksnap.org). The cystic change, necrosis, and calcification regions were carefully excluded to obtain a more homogenized dataset (12). All the ROIs were confirmed by another senior radiologist with more than 20 years of experience in head and neck imaging. Both radiologists were blind to the pathological results.

### Data Pretreatment for the DL Models

Before the development of the DL models, each manually labeled ROI was transformed and defined as follows: (i) a three-dimensional (3D) patch of 96*96*16 pixels containing the cropped lymph nodes region, whose size was determined based on the largest ROI; (ii) tumor masks, in which non-lesion areas were left padded with zero, were manually labeled pixelwise; and (iii) the pathologically identified label of tumor metastasis.

Owing to the limited amount of training data, we also used data augmentation approaches, including flipping (perpendicular to the x and y axes), random rotation (90, 180, and 270 degrees perpendicular to the z axis), and random brightness contrast (80%, 90%, 110% and 120%) in the development set. After data augmentation, the sample size increased to 10 times that of the original, yielding a total of 2340 samples for the development of the DL models.

### Development of the Deep Learning Models

**Figure 2:**
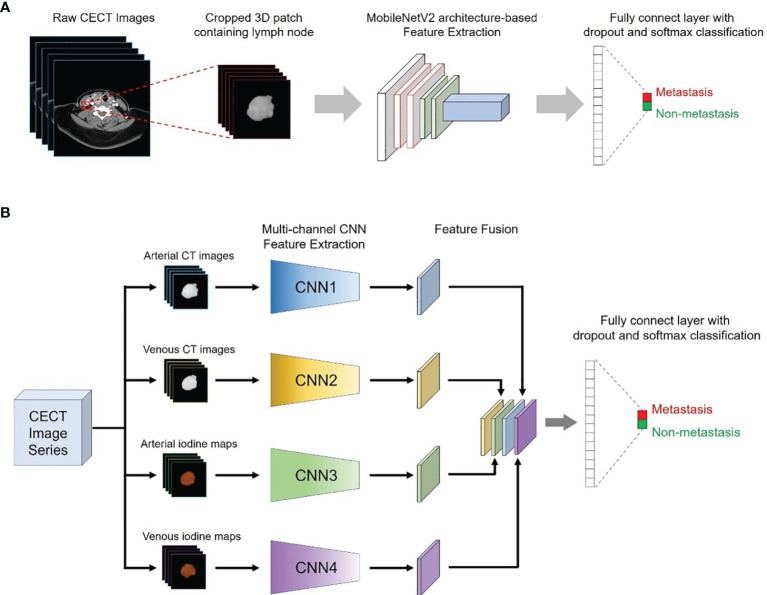
Conceptual architecture of the single-modality DL model **(A)** and the multichannel DL model integrating all ROIs from the CT images and iodine maps **(B)**.

The MobileNetV2 network was used as the backbone structure of the DL model due to the faster calculation speed than other classic neural networks (e.g., IncePtionV3 and VGG16) while maintaining similar accuracy and greatly simplifying the number of parameters, which reduces the risk of overfitting for small sample sizes. In addition, the original MobilenetV2 model was entered into a 3D version according to the data characteristics in this study. More details about the modification of the 3D MobileNetV2 network were presented in the **Supplementary Data**. Two kinds of DL models were proposed in this study: a single-channel neural network for each modality of the DECT images (arterial phase CT images, venous phase CT images, arterial iodine maps and venous iodine maps) and a multichannel neural network that integrated four modalities of the DECT images (**Figure 2**).

Therefore, a total of five DL models were proposed in our study: four single-modality DL models based on arterial phase CT images (CT-A model), venous phase CT images (CT-V model), arterial iodine maps (Iodine-A model) and venous iodine maps (Iodine-V model), and a multichannel DL model using ROIs from all modalities as input (Combined model).

To improve the robustness of the model and achieve better performance, transfer learning methods were also applied. The neural networks used in this study were first pretrained on natural images from The ImageNet natural image dataset and were then pretrained on multiple medical image datasets from The Cancer Imaging Archive (TCIA) database.

**Figure 3:**
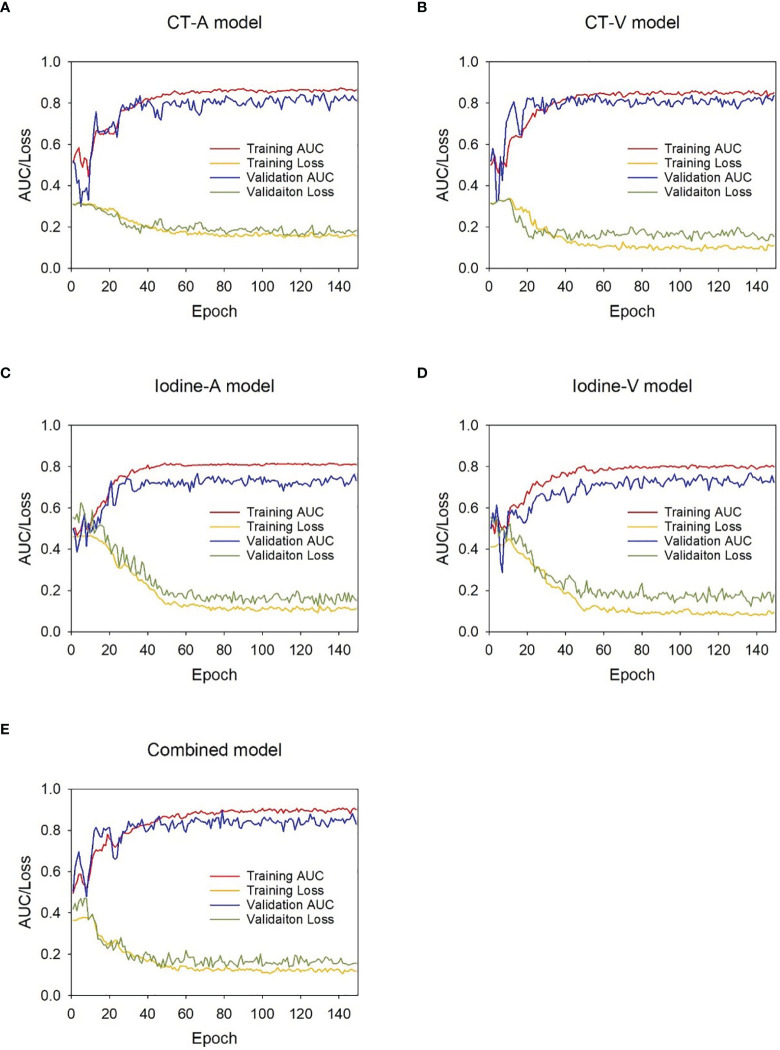
AUC-loss curve of the DL models during development stage. **(A)** CT-A model, **(B)** CT-V model, **(C)** Iodine-A model, **(D)** Iodine-V model ,**(E)** Combined model.

The proposed DL models were trained based on the binary cross-entropy loss function, which is commonly used for classification tasks. Adam was used as the optimizer in the training stage owing to its fast convergence and weight-dependent learning rate. The initial learning rate and the weight decay were set to 0.0001 and 0.01, respectively. The minibatch size was set to 24, and the dropout rate was set to 0.5. The weight parameters of the initialized hidden layer were randomly allocated, and other parameters were set as default. During model development, 5-fold cross-validation was applied to avoid overfitting, and the weighted ensemble method was applied to integrate a weighted average result from those cross-validation models. The training was stopped when the loss function was stable. The relationship between the model efficiency (AUC) and the cross-entropy loss function index at each epoch during the model development process is presented in **Figure 3**.

The supervised training process of the DL models was performed on the InferScholar platform version 3.5 (InferVision, China) with a Core i7-7700 K central processing unit (Intel, Santa Clara, Calif), 32 GB memory, and a GeForce GTX 2070 graphics processing unit (NVIDIA, Santa Clara, Calif). Python 3.6.8 (https://www.python.org) and the Mxnet 1.5.0 framework for neural networks (https://mxnet.incubator.apache.org) were used to construct the DL models. The code of the DL models was available at https://github.com/Sarah-huiling/DL_ThyroidLymphNode.git.

### Performance Evaluation of the Predictive Models

The discriminative efficacy of the DL models was evaluated by receiver operating characteristic (ROC) analysis with respect to the area under the curve (AUC). In addition, the sensitivity, specificity, positive predictive value (PPV) and negative predictive value (NPV) of each model were also calculated under the optimal threshold according to the maximum Youden index (13).

### Calibration and Decision Curve Analysis

The consistency between the predicted metastasis probability and actual metastasis rate was evaluated through calibration curves using the 1,000 bootstrapping resamples method, and the Hosmer–Lemeshow test was conducted to assess the goodness-of-fit of the predictive models in both the development and independent testing sets (14). Decision curve analysis (DCA) was used to assess the clinical utility of the predictive models by estimating the net benefits for a range of threshold probabilities in the independent testing set (15).

### Performance Comparison Between Artificial Intelligence (AI) and the Radiologists

We used the independent testing set to compare the diagnostic performance of the AI (the combined model) with that of 4 radiologists (2 senior radiologists with 15 and 12 years of experience and 2 junior radiologists with 4 and 5 years of experience). To evaluate the actual impact of the DL model in clinical practice, all radiologists first diagnosed the lymph nodes in the independent testing set independently, and they were asked to diagnose the same lymph nodes again with AI assistance after a washout period of 4 weeks. All the radiologists were aware that the cases in the independent testing set had undergone biopsy or surgery, but they were blinded to the pathological reports.

### Statistical Analysis

Statistical analyses were performed using SPSS software (version 23.0) and MedCalc software (version 20.0). Continuous variables were compared by Student’s t-test or the Mann–Whitney U test, and categorical variables were compared by the chi-square test or Fisher’s exact test, where appropriate. The difference between two AUCs of different models was compared with Delong’s test (16) or the Hanley-McNeil test (17), where appropriate. The weighted kappa value was used to assess the interobserver agreement of the two radiologists. The calibration curve was plotted using the “rms” package, and the decision curve was plotted using the “rmda” package. A two-sided p < 0.05 was considered statistically significant.

## Results

### Patient Characteristics

A total of 319 patients from April 2018 to December 2020 in our hospital were initially recruited. According to the inclusion and exclusion criteria, 22 men (mean age, 40.9 ± 14.6 years) and 56 women (mean age, 40.7 ± 11.9 years) were enrolled in the study. There was no significant difference in the prevalence of lymph nodes metastasis (chi-square test, p = 0.868) between the development set (40.2%, 94/234) and the independent testing set (39.0%, 23/59).

### Analysis of the CT Image Features

As shown in **Table 1**, the CT image features were compared between nonmetastatic and metastatic lymph nodes in the development set and independent testing set. Shape, enhancement degree and enhancement pattern were selected through multivariate logistic regression analysis (**Supplementary Table 1**), and the prediction value of the CT-feature model was calculated using following formula:


Predictionvalue=−1.5411+1.08356×Shape(Regular=0,Irregular=1)+1.19649×Enhancementpattern(Homogeneous=0,Heterogeneous=1)+0.90834×Enhancementdegree(Mild-moderate=0,Strong=1).

**Table 1:** Comparison of CT image features between no-metastatic and metastatic lymph nodes.

CT image features	Development set			Independent testing set		
	Nonmetastatic (n=140)	Metastatic (n=94)	*p*	Nonmetastatic (n=36)	Metastatic (n=23)	*p*
Size			*0.549*			*0.735*
5~10 mm	109	70		25	15	
>10 mm	31	24		11	8	
Shape			*<0.001*			*0.250*
Regular	117	55		27	14	
Irregular	23	39		9	9	
Margin			*0.075*			*0.071*
Clear	115	68		27	12	
Unclear	25	26		9	11	
Enhancement degree			*<0.001*			*0.006*
Mild-moderate	94	36		21	5	
Strong	46	58		15	18	
Enhancement pattern			*<0.001*			*0.006*
Homogeneous	112	45		27	9	
Heterogeneous	28	49		9	14	
Calcification			*0.005*			*0.010*
Yes	3	10		0	4	
No	137	84		36	19	
Cystic change			*<0.001*			*0.313*
Yes	0	14		1	2	
No	140	80		35	21	
Extra-nodal extension			*0.001*			*0.207*
Yes	0	7		0	1	
No	140	87		36	22	

### Performance Evaluation of the Predictive Models

**Figure 4:**
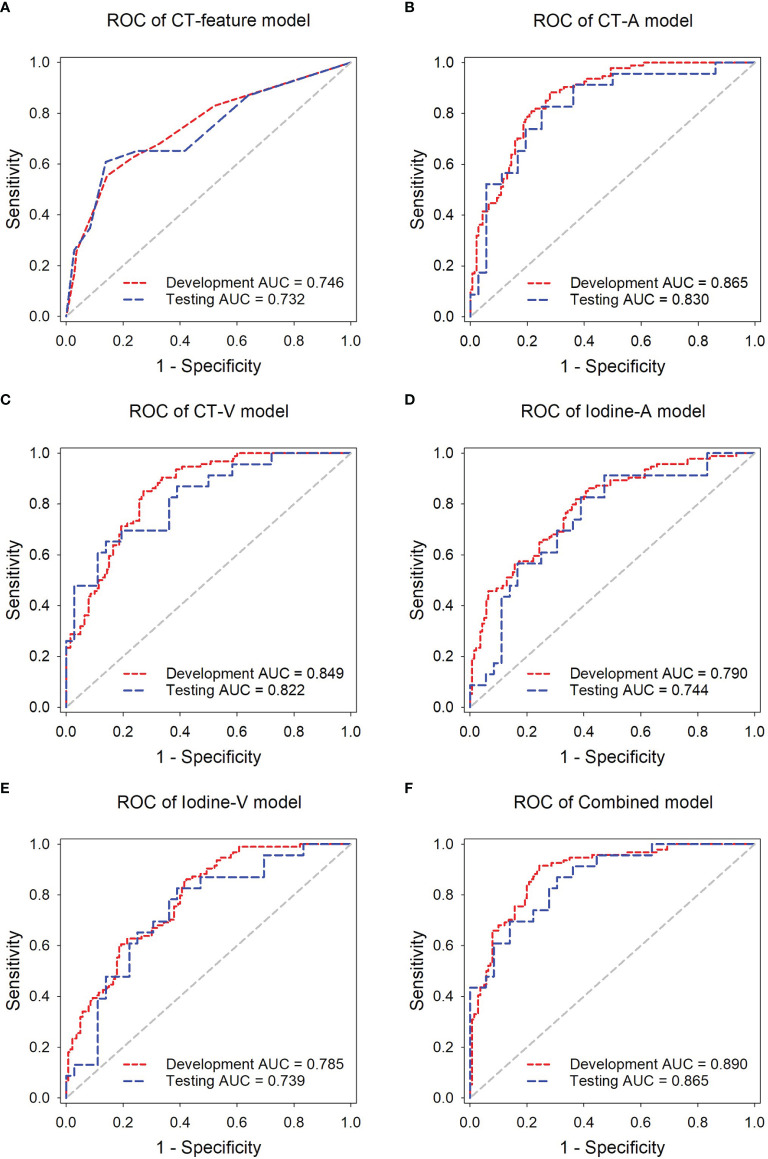
ROC analysis of the predictive models in the development and independent testing sets. ROC curves of the **(A)** CT-feature model, **(B)** CT-A model, **(C)** CT-V model, **(D)** Iodine-A model **(E)** Iodine-V model, and **(F)** Combined model in the delelopment and testing sets, respectively.

The ROC analysis of the predictive models in the development and independent testing sets was shown in **Figure 4**. The AUCs of the CT-feature model were 0.746 (95% CI, 0.685~0.800) in the development set and 0.732 (95% CI, 0.601~0.839) in the independent testing set. The CT-A, CT-V, Iodine-A, and Iodine V models achieved AUCs of 0.865 (95% CI, 0.814~0.906), 0.849 (95% CI, 0.797~0.892), 0.791 (95% CI, 0.733~0.841) and 0.785 (95% CI, 0.727~0.836) in the development set, and the AUCs of these models were 0.830 (95% CI, 0.709~0.915), 0.822 (95% CI, 0.701~0.910), 0.744 (95% CI, 0.614~0.849) and 0.739 (95% CI, 0.608~0.845) in the testing set, respectively. In general, the CT image-based models showed higher performance than the iodine map-based models; Only one group was significantly different (CT-A model *vs*. Iodine-A model, p = 0.043 in the development set), with no significant differences seen in the remaining three groups (CT-A model *vs*. Iodine-A model, p = 0.151 in the independent testing set; CT-V model *vs*. Iodine-V model, p = 0.074 in the development set and p = 0.273 in the independent testing set). There were no significant differences between the arterial phase image-based models and venous phase image-based models (CT-A model *vs*. CT-V model, p = 0.655 in the development set and p = 0.870 in the independent testing set; Iodine-A model *vs*. Iodine-V model, p = 0.889 in the development set and p = 0.957 in the independent testing set).

The Combined model, incorporating both arterial and venous phase CT images and the corresponding iodine maps, showed the highest accuracy in predicting lymph nodes metastasis, with AUCs achieving 0.890 (95% CI, 0.842~0.927) in the development set and 0.865 (95% CI, 0.751~0.940) in the independent testing set. The AUCs of the Combined model were significantly higher than those of the Iodine-A model (p = 0.007), Iodine-V model (p = 0.004) and CT-feature model (p < 0.001) in the development set. Although not statistically significant, the Combined model showed better performance than the CT-A model (p = 0.427) and CT-V (p = 0.225) in the development set and all the other predictive models in the independent testing set (p = 0.096 *vs*. the CT-feature model, p = 0.330 *vs*. the CT-A model, p = 0.237 *vs*. the CT-V model, p = 0.074 *vs*. the Iodine-A model, p = 0.085 *vs*. the Iodine-V model). The detailed sensitivity, specificity, PPV and NPV of these models in the development set and the independent testing set are summarized in **Tables 2**, **3** respectively. In addition, subsequent ROC analysis according to the size of lymph nodes (5~10 mm or >10mm) was performed (**Supplementary Figure 1**), and the performance of the Combined model was consistent across lymph node size (**Supplementary Table 2**).

**Table 2:** Performance comparison of different models in the development set.

Model	AUC (95% CI)	*p-value*	Threshold	Sensitivity	Specificity	PPV	NPV
CT-feature	0.746 (0.685~0.800)	*<0.001*	>0.3753	55.3%	85.7%	72.2%	74.1%
CT-A	0.865 (0.814~0.906)	*0.427*	>0.4518	88.3%	72.1%	68.0%	90.2%
CT-V	0.849 (0.797~0.892)	*0.225*	>0.4202	85.1%	72.9%	67.8%	87.9%
Iodine-A	0.791 (0.733~0.841)	*0.007*	>0.3491	81.9%	62.9%	59.7%	83.8%
Iodine-V	0.785 (0.727~0.836)	*0.004*	>0.4097	86.2%	57.9%	57.9%	86.2%
Combined	0.890 (0.842~0.927)	*reference*	>0.4239	91.5%	75.7%	71.7%	93.0%

**Table 3:** Performance comparison of different models in the independent testing set.

Model	AUC (95% CI)	*p-value*	Threshold	Sensitivity	Specificity	PPV	NPV
CT-feature	0.732 (0.601~0.839)	*0.096*	>0.6248	60.9%	86.1%	73.7%	77.5%
CT-A	0.830 (0.709~0.915)	*0.330*	>0.3818	82.6%	75.0%	67.9%	87.1%
CT-V	0.822 (0.701~0.910)	*0.237*	>0.6009	65.2%	86.1%	75.0%	79.5%
Iodine-A	0.744 (0.614~0.849)	*0.074*	>0.3785	91.3%	52.8%	55.3%	90.5%
Iodine-V	0.739 (0.608~0.845)	*0.085*	>0.3473	82.6%	61.1%	57.6%	84.6%
Combined	0.865 (0.751~0.940)	*reference*	>0.4387	87.0%	69.4%	64.5%	89.3%

### Calibration and Clinical Utility Analysis

**Figure 5:**
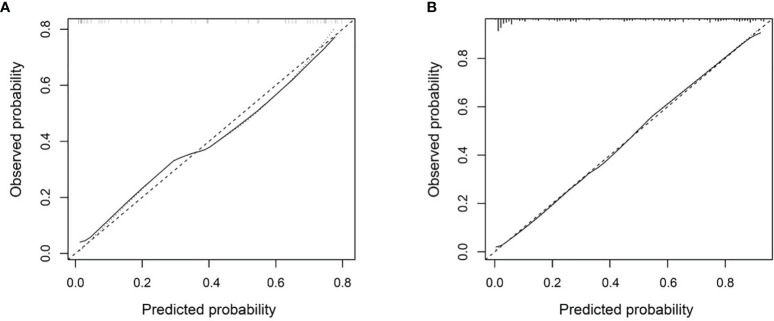
Calibration curve of the Combined model in the development set **(A)** and the independent testing set **(B)**. The X axis and Y axis represent the predicted lymph nodes metastasis probability and the actual metastasis rate, respectively. A closer fit to the diagonal gray dash line represents a better prediction.

The Combined model showed good consistency between the predicted lymph nodes metastasis probability and the actual metastasis rate in both the development and independent testing sets (**Figure 5**). The calibration curve suggested no significant deviation from an ideal fitting, with the nonsignificant statistic of the Hosmer–Lemeshow test achieving p = 0.070 and 0.803 in the development and independent testing sets, respectively.

**Figure 6:**
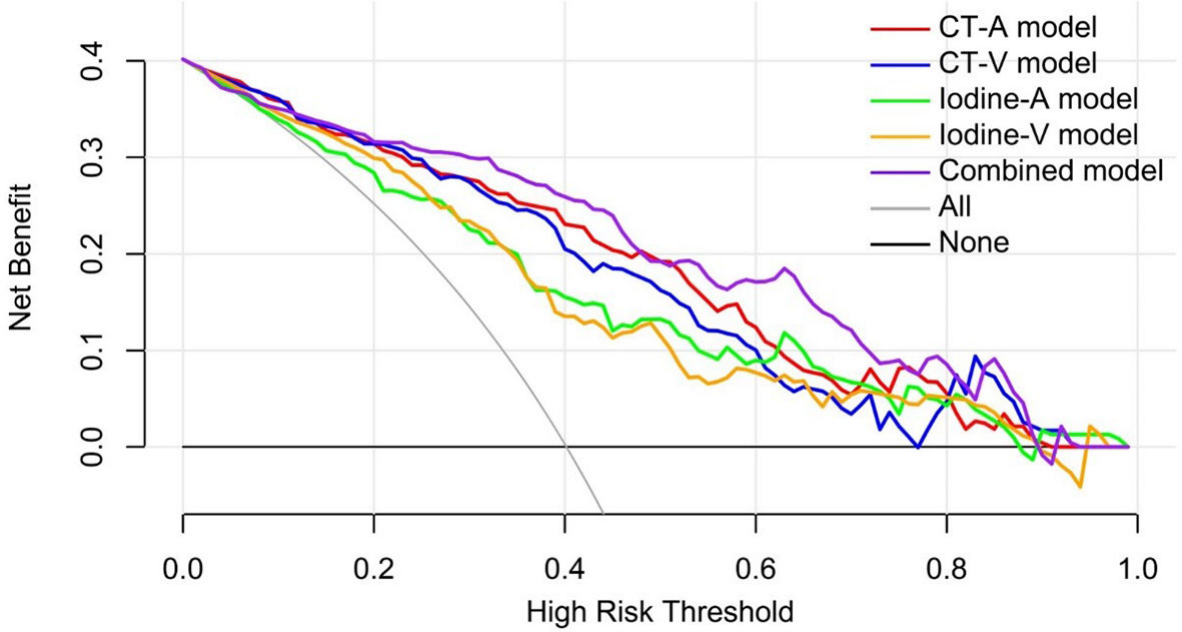
Decision curve analysis for the predictive models in the independent testing set. The gray line and black line represented situations in which all lymph nodes were metastatic and no lymph nodes were free of cancer, respectively.

The decision curve analysis for the DL models in the independent testing set indicated that the Combined model showed a higher overall net benefit in differentiating metastatic LNs from nonmetastatic LNs than other single-modality DL models, which demonstrated the superiority of the Combined model compared with other models in terms of clinical usefulness (**Figure 6**).

### Comparison of the Combined Model and Radiologists in the Independent Testing Set

**Figure 7:**
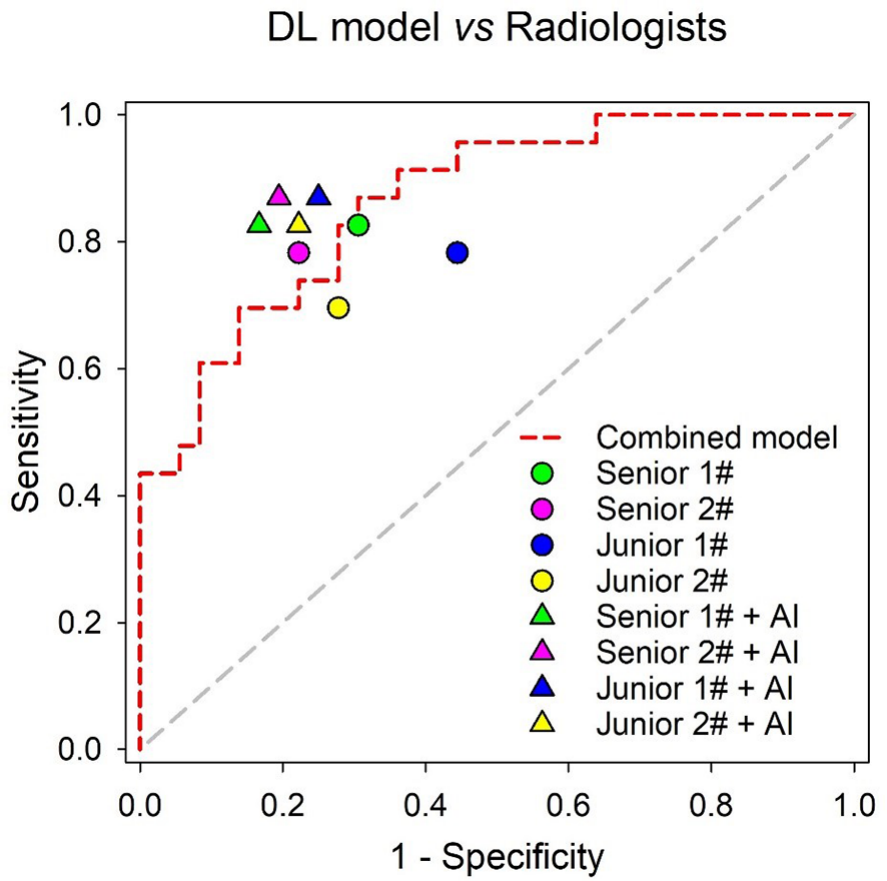
Performance comparison of the Combined model and radiologists in the independent testing set.

Although not statistically significant, the Combined model achieved a higher AUC than senior radiologist #1 (p = 0.180) and senior radiologist #2 (p = 0.262), while it significantly outperformed the two junior radiologists (both p values < 0.05) in the independent testing set. All radiologists benefited from AI assistance, with the AUCs of the two senior radiologists increasing from 0.760 to 0.830 (p = 0.359) and 0.780 to 0.838 (p = 0.335). The AUCs of the two junior radiologists also increased from 0.669 to 0.810 (p = 0.020) and 0.709 to 0.802 (p = 0.258), respectively. In addition, the interobserver agreement between the two junior radiologists also improved after AI assistance, with a weighted kappa of 0.334 (95% CI, 0.104~0.564) increasing to 0.593 (95% CI, 0.387~0.798). The results of the ROC analysis are presented in **Figure 7**, and the detailed sensitivity, specificity, PPV, NPV, and AUC are summarized in **Table 4**.

**Table 4:** Comparison of the diagnostic efficiency between the Combined model and the radiologists (with or without AI assistance) in the independent testing set.

	AUC (95% CI)	*p-value*	Sensitivity	Specificity	PPV	NPV
Senior Radiologist 1	0.760 (0.631~0.862)	*0.180*	82.6%	69.4%	63.3%	86.2%
Senior Radiologist 2	0.780 (0.653~0.878)	*0.262*	78.3%	77.8%	69.2%	84.8%
Junior Radiologist 1	0.669 (0.534~0.786)	*0.021*	78.3%	55.6%	52.9%	90.0%
Junior Radiologist 2	0.709 (0.576~0.820)	*0.043*	69.6%	72.2%	61.5%	78.9%
Senior Radiologist 1 + AI	0.830 (0.709~0.915)	*0.532*	82.6%	83.3%	76.0%	88.2%
Senior Radiologist 2 + AI	0.838 (0.718~0.921)	*0.680*	87.0%	80.6%	74.1%	90.6%
Junior Radiologist 1 + AI	0.810 (0.687~0.900)	*0.438*	87.0%	75.0%	69.0%	90.0%
Junior Radiologist 2 + AI	0.802 (0.678~0.894)	*0.202*	82.6%	77.8%	70.4%	87.5%
Combined model *	0.865 (0.751~0.940)	*reference*	87.0%	69.4%	64.5%	89.3%

^*^The sensitivity, specificity, PPV and NPV of the Combined model were calculated under the optimal cut-off point. (threshold = 0.4387) when compared with that of the radiologists.

## Discussion

In this study, we developed five deep learning models based on multiphase dual-energy spectral CT to predict lymph nodes metastasis preoperatively and noninvasively in papillary thyroid cancer patients. Using the independent testing set, our study showed that the combined DL model possessed superior diagnostic capability compared to that of the other four single-modality DL models, with AUCs of 0.865. Meanwhile, the combined DL model showed high diagnostic efficacy across the size of LNs with AUCs achieving 0.845,0.898 in LNs of 5-10mm and >10mm, respectively. Although not statistically significant, we found that single-modality DL models based on the arterial phase achieved a better sensitivity and diagnostic performance whether CT images or iodine maps were used. The results were consistent with previously published studies, and these studies considered that arterial phase CT could highlight the difference between metastatic and benign LNs because tumor angiogenesis and recruitment of capsular vessels increased tumor perfusion in metastatic LNs (18).

The thyroid gland is the main organ with the capacity for iodine intake in the human body, and metastatic LNs from PTC can take up iodine. In addition, increased tumor vascularity in metastatic LNs may contribute to an increase in iodine uptake (1, 19). Hence, the utilization of iodine maps for the evaluation of metastatic LNs from PTC has certain advantages. However, the results from our study showed inconsistency with the above theory, which found that the iodine map-based models were not better than the CT image-based models, regardless of arterial phase or venous phase. The reasons for the inconsistency of the results may be that although iodine maps can highlight the difference of iodine intake in tissues, it is inferior to conventional CT image in the display of morphological features, such as boundary and internal structure. Nevertheless, the DL models based on iodine maps exhibited relatively high sensitivity (91.3% of the iodine-A model, 82.6% of the iodine-V model) in the independent testing set.

The conventional approach to diagnose metastatic cervical LNs is according to the morphologic characteristics of the nodes, including size or shape, central necrosis or cystic degeneration, degree and pattern of enhancement and extra-nodal extension (20). This approach is not only controversial but also challenging, especially for unskilled radiologists. The current studies could not come to an agreement. Zhou et al. indicated that morphological features larger than 10 mm in size, with irregular shape, unclear margins, strong enhancement, heterogeneous enhancement, and extra-nodal extension were highly suggestive of metastatic LNs (19). However, Liu et al. reported that the degree and pattern of enhancement were only valuable indicators for differentiating LNs status (1). Moreover, a study from J.E. Park et al. reported that the method for the detection of cervical LNs using morphologic characteristics had relatively low sensitivity, with a value of only 46.8% (9).Our study found that the degree and pattern of enhancement and calcification showed significant differences both in development and independent testing set. but, the shape, cystic change, and extra-nodal extension showed significant differences only in development set. The reasons for the results may be that the sample size of independent testing set was not large enough to reflect the difference. The result also manifested that evaluating LNs by the morphological features was controversial. In addition, our study found the Combined model had better performance than the CT-feature model but there was significant difference only in the development set. This reason may be that the CT features were evaluated by senior radiologists, and the Combined model did not include CT-feature model, which was in accordance with the result of comparison between the Combined model and the senior radiologists.

The results of this study showed that the Combined model significantly outperformed two junior radiologists (both p values < 0.05) and showed noninferior diagnostic capability compared with the senior radiologists in the independent testing set. Notably, our study found that all radiologists benefited from AI assistance, the junior radiologists received more help from AI assistance than the junior radiologists, and the interobserver agreement between the two junior radiologists significantly increased from a kappa of 0.334 to 0.593. Our findings were consistent with those of the recent work by Lee et al., which demonstrated that deep learning-based computer-aided diagnosis could help resident physicians gain confidence in diagnosis, improve diagnostic accuracy and increase overall confidence levels for CT diagnosis of cervical LNs metastasis from thyroid cancer using a large clinical cohort (21).

Some limitations of this study should be noted. First, our dataset was obtained from a single center, and all patients underwent unified contrast-enhanced CT protocols, which can cause biases. Further study will require a larger sample size from multiple centers to validate our results and increase their repeatability. Second, the deep learning model was not combined with clinical data, which should be incorporated in further studies. Third, this study only excluded lymph nodes less than 5mm to avoid a partial volume effect. However, malignant nodes smaller than 5 mm are often encountered. Further studies need to improve the ability of LNs segmentation to include smaller lymph nodes, which will be better able to reflect reality.

In conclusion, a Combined model integrating both CT images and iodine maps of the arterial and venous phases showed good performance in predicting lymph nodes metastasis in thyroid cancer patients, which could facilitate clinical decision-making.

## Data Availability Statement

The raw data supporting the conclusions of this article will be made available by the authors, without undue reservation.

## Ethics Statement

This study was approved by the Institutional Ethics Committee of the Second Affiliated Hospital of Soochow University, and the written informed consent requirement was waived.

## Author Contributions

DJ, XN, and XZ have contributed equally to this work and share first authorship. DJ, XN, and XZ: collected and analyzed the data, plotted tables, and wrote this article. HY: analyzed the results. HZ: suggested Deep Learning approaches. LX: provided medical advice. RW: collected and analyzed the data. GF: designed and implemented the work and provided medical advice. All authors contributed to manuscript revision, read, and approved the submitted version.

## Funding

This work was supported by the Suzhou Science & Technology Innovation Project (Grant No. ZHYLYB 2021001).

## Conflict of Interest

HY and HZ were employed by Infervision Medical Technology Co., Ltd, Beijing, China.

The remaining authors declare that the research was conducted in the absence of any commercial or financial relationships that could be construed as a potential conflict of interest.

## Publisher’s Note

All claims expressed in this article are solely those of the authors and do not necessarily represent those of their affiliated organizations, or those of the publisher, the editors and the reviewers. Any product that may be evaluated in this article, or claim that may be made by its manufacturer, is not guaranteed or endorsed by the publisher.
