# Supplementary material for: Multiphase Dual-Energy Spectral CT-Based Deep Learning Method for the Noninvasive Prediction of Head and Neck Lymph Nodes Metastasis in Patients With Papillary Thyroid Cancer
Source: Front Oncol. 2022 Apr 20;12:869895. doi: 10.3389/fonc.2022.869895 (PMC9065438; doi:10.3389/fonc.2022.869895)
Supplement: Supplementary file 1 [file DataSheet_1.docx]

**supplementary**

***Modification of the 3D version MobileNetV2 network***

MobileNetV2 was used as the backbone network of the proposed deep learning models in this study. The original MobilenetV2 network was modified into a 3D version because of the cropped 3D patches from dual-energy CT images. In brief, the architecture of the modified 3D MobilenetV2 model was consisted of four MobilenetV2 3D blocks with spatiotemporal down-sampling (2×) and nine MobilenetV2 3D blocks. The architecture of the modified 3D MobileNetV2 model was shown in our previous work[1]

[1] Zhang W, Yin H, Huang Z, Zhao J, Zheng H, He D, Li M, Tan W, Tian S, Song B. **Development and validation of MRI-based deep learning models for prediction of microsatellite instability in rectal cancer**. *Cancer Med* 2021,**10**(12):4164-4173


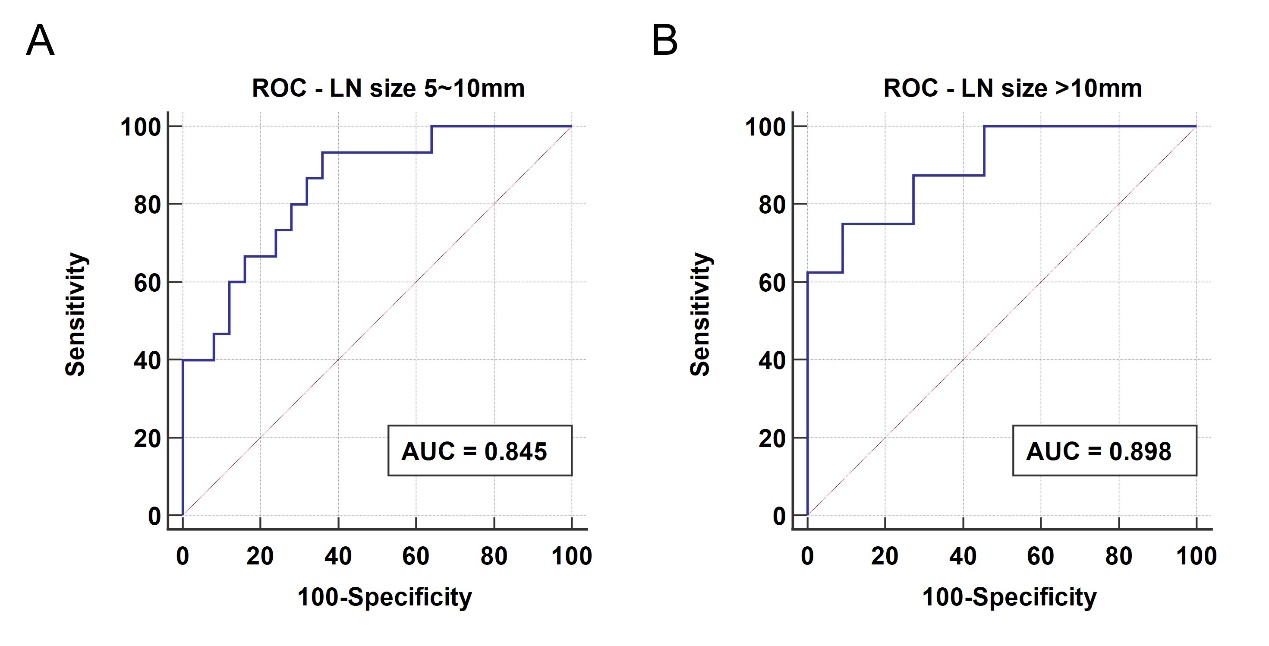


**Supplementary Figure 1**. ROC analysis of the Combined model in the independent testing set across the size of lymph nodes. (A) Lymph node size 5~10 mm. (B) Lymph node size >10 mm.

**Supplementary Table 1**. Multivariate logistic regression analysis of the CT image features in the development set.

| Features | Odds ratio (95% CI) | p-value | Coefficient | Intercept |
| --- | --- | --- | --- | --- |
| Shape | 2.9552 (1.3774~4.4661) | 0.0011 | 1.08356 |  |
| Enhancement pattern | 3.3085 (1.7890~6.1186) | 0.0001 | 1.19649 |  |
| Enhancement degree | 2.4802 (1.3774~4.4661) | 0.0025 | 0.90834 |  |
|  |  |  |  | -1.5411 |

**Supplementary Table 2.** Performance of the Combined model across lymph node size in the independent testing set.

| LN size | AUC (95% CI) | Threshold | Sensitivity | Specificity | PPV | NPV |
| --- | --- | --- | --- | --- | --- | --- |
| 5~10 mm | 0.845 (0.696~0.940) | >0.3965 | 93.3% | 64.0% | 60.9% | 94.1% |
| >10 mm | 0.898 (0.672~0.988) | >0.4329 | 87.5% | 72.7% | 70.0% | 88.9% |
